# Transcriptome analysis of salt-responsive and wood-associated NACs in *Populus simonii × Populus nigra*

**DOI:** 10.1186/s12870-020-02507-z

**Published:** 2020-07-06

**Authors:** Wenjing Yao, Chuanzhe Li, Shuyan Lin, Jianping Wang, Boru Zhou, Tingbo Jiang

**Affiliations:** 1 State Key Laboratory of Tree Genetics and Breeding, Northeast Forestry University, 51 Hexing Road, Harbin, 150040 China; 2 Co-Innovation Center for Sustainable Forestry in Southern China/Bamboo Research Institute, Nanjing Forestry University, 159 Longpan Road, Nanjing, 210037 China; 3 Institute of Agricultural Resources and Environment, Jiangsu Academy of Agricultural Sciences, Nanjing, 210014 China; 4 Department of Agronomy, University of Florida, 2033 Mowry Road, Gainesville, FL32610 USA

**Keywords:** *Populus simonii×P. nigra*, NAC transcription factor, Salt stress, Transcriptome analysis, Phylogenic analysis

## Abstract

**Background:**

NAC (NAM, ATAF1–2, and CUC2) family is one of the largest plant-specific transcription factor families known to play significant roles in plant development processes and stress responses.

**Results:**

In the study, a total of 112 NACs were identified to be differentially expressed in the comparisons of leaves and stems, leaves and roots, roots and stems of *Populus simonii×P. nigra* among 289 members by RNA-Seq. And 148, 144 and 134 NACs were detected to be salt-responsive in the roots, stems and leaves under 150 mM NaCl stress, respectively. Among them, a total of 53 salt-responsive NACs were shared across the three tissues. Under salt stress, 41/37 NACs were identified to be up/down-regulated in the leaves of *Populus simonii × P.nigra* among 170 non-redundant NACs by RT-qPCR, which was similar with RNA-Seq results. The expression pattern analysis of 6 NACs including four randomly up-regulated genes (*NAC86*, *NAC105*, *NAC139* and *NAC163*) and two down-regulated genes (*NAC15* and *NAC149*) indicated a few NACs showed specific temporal and spatial expression patterns in the three tissues of *Populus simonii×P.nigra*. Based on transcriptome screening and phylogenic analysis of differentially expressed NACs in different tissues under salt stress, 18 potential NACs associated with wood formation and 20 involved in stress responses were identified in *Populus simonii×P.nigra*.

**Conclusions:**

The study further gains an understanding of the connection of tissue specificity and gene function in poplar, and lays the foundation of functional analysis of poplar NACs in stress responses.

## Background

NAC was originally derived from the names of three proteins, NAM, ATAF1–2, and CUC2 [1]. NAC family is probably the largest plant-specific transcription factor (TF) family with approximately 20,000 members [2]. All the members in this family share a highly conserved N-terminal NAC domain with 150–160 amino acids. The domain can be divided into five sub-domains (A-E). The sub-domains A, C, and D are highly conserved, while B and E are variable. The A sub-domain is associated with the formation of functional dimer. The C and D sub-domains with positive charge are important DNA-binding regions. The B and E sub-domains may be involved in functional diversity of NAC proteins [3]. NAC family has diversified C-terminal transcriptional regulatory regions with several specific motifs that are rich in repeats of serine-threonine, proline-glutamine, or acidic residues, which function as either transcriptional activators or repressors [3, 4]. Act as central regulators in plant developmental processes and various stress responses, a few NAC TFs can interact with downstream genes by specifically binding to cis-acting elements in the promoter regions of target genes. Some significant elements are ABREs, DREs, LTREs, MYB, MYC etc. [4, 5]. In addition, a few researches indicated several upstream elements and *miRNAs* play an important role in regulating the expression of NAC proteins in plant development and environment stress responses [6, 7]. For instance, MITE element was proved to be inserted in the promoter of *ZmNAC111* and significantly associated with natural variation in maize drought tolerance [6]; a few *miRNAs* such as *miRNA164*, *miRNA159*, *miRNA166*, *miRNA319*, etc. serve as key players in the overlapping genetic networks by regulating the expression of NAC proteins associated with plant growth and stress responses [7].

NACs can regulate multiple biological processes, including plant developmental processes, hormones biosynthesis, metabolism processes, stress responses etc. [8]. NACs participate in multiple plant development processes such as lateral root development, organs formation, cell division and differentiation, plant senescence, and secondary cell wall formation [9–12]. Specifically, certain NACs act as master gene regulators during wood formation, such as vascular-related NAC-domain (*VND*) genes, secondary wall-associated NAC domain (*SND*) genes, and NAC secondary wall thickening (*NST*) genes [13–16]. For example, *VND7* participates in wood formation by directly regulating the expression of genes involved in xylem vessel formation [14]; *SND1* can regulate secondary wall synthesis in fibers of *Arabidopsis* [15]; *NST1* and *NST2* can regulate secondary wall thickening in *Arabidopsis* [16]. In addition, a few NACs play an important role in plant hormones biosynthesis, reception and signaling regulation [17]. Furthermore, several NACs are associated with reactive oxygen species accumulation and cell death [18]. Last but not least, a number of NACs have been identified to participate in biotic/abiotic stress responses [4, 5].

Considerable effort has been made to reveal regulatory function of NACs in stress responses [19]. NACs function in multiple stress responses via auto-regulation or cross-regulation of stress-related genes in various signaling pathways and regulatory networks [20]. Taken *ANAC096* as an example, it cooperated with bZIP-type ABRE binding factor and ABRE binding protein to improve plant stress tolerance under dehydration and osmotic stress conditions [21]. Most NACs are positive regulators in plant stress responses. For instance, *TaNAC2* enhanced tolerances to drought, salt, and freezing stresses in transgenic *Arabidopsis* [22]. *SlNAC4* acted as a stress-responsive TF in positive modulation of salt and drought tolerance in tomato [23]. Also there are several NACs playing opposite roles in stress tolerance. For example, a novel NAC gene from *Arabidopsis*, *ATAF1*, was drought-inducible and negatively regulated the expression of stress-responsive genes [24]. *ONAC095* was identified to function as a negative regulator in drought and cold stress responses in transgenic rice [25]. In addition, a few NACs are involved in crosstalk between abiotic and biotic stress signaling. For instance, *OsNAC6* improved tolerance to dehydration and high salt, as well blast disease in transgenic rice [26]. Transgenic *Arabidopsis* overexpressing *ATAF1* enhanced drought tolerance, but was highly sensitive to necrotrophic fungus [27]. Additionally, certain NACs may function in both plant response to environmental stresses and modulation of plant development processes. *GmNAC20* from soybean not only enhanced salt and freezing tolerance, but also promoted lateral root formation in transgenic *Arabidopsis* [28]. *Arabidopsis AtNAP* was identified to be a negative regulator in osmotic stress responses and a positive regulator in senescence [29].

With the population of next-generation sequencing (NGS), the increasing studies about genome-wide analyses of NAC family of various crops such as buckwheat, sorghum, millet, rice, soybean, potato etc. emerged in recent years [30–36]. For example, a total of 80 *FtNACs* were obtained from buckwheat on a genome-wide basis [30]; as many as 145 non-redundant *SbNACs* were identified from sorghum by genome-wide survey [31]; gene structures, phylogenies, genome localizations, and expression profiles of 151 non-redundant NAC genes from rice were investigated through complete genome-wide overview [32]; 147 putative NACs were identified from foxtail millet by comprehensive genome-wide analyses and genomic constitution [33]. In addition, downstream genes of NACs can also be identified by genome-wide association study. For instance, the direct target genes of OsNAC proteins were identified using RCc3:6MYC-OsNAC expressing roots of transgenic rice by ChIP-Seq and RNA-Seq [34]. However, the genome-wide analyses of NAC family in trees are much less than those in crops.

*Populus* is a model tree plant for biology study [37]. Di-haploid *Populus simonii×P. nigra* is a specific hybrid *Populus* and plays an important role in economic and shelter forest construction in the northeast, northwest and southwest of China [38]. Understanding regulatory roles of NACs in wood formation and stress responses will contribute to timber quality and stress tolerance of *Populus simonii×P. nigra*. Therefore, we focused on profiling and screening of salt-responsive and wood-associated NACs in *Populus simonii×P. nigra* by transcriptome analysis in the study. Firstly, differentially expressed genes (DEGs) and 289 NAC members were profiled and screened in the leaves, stems and roots of *Populus simonii×P. nigra* by RNA-Seq. Then salt-responsive DEGs and NACs were identified in the three tissues, respectively. Particularly the relative expression level of 170 non-redundant NACs was quantified in the leaves of *Populus simonii×P. nigra* under salt stress by RT-qPCR. And the expression pattern of 6 NACs under salt stress at different time point was validated in the three tissues by RT-qPCR. In addition, transcriptome profiling and phylogenic analysis were combined for identifying potential poplar NACs associated with wood formation and salt stress response. The results present in the study shed light on functional characterization of NACs in poplar.

## Results

### The DEGs in the comparisons among the three tissues of *Populus simonii×P. nigra*

**Fig. 1:**
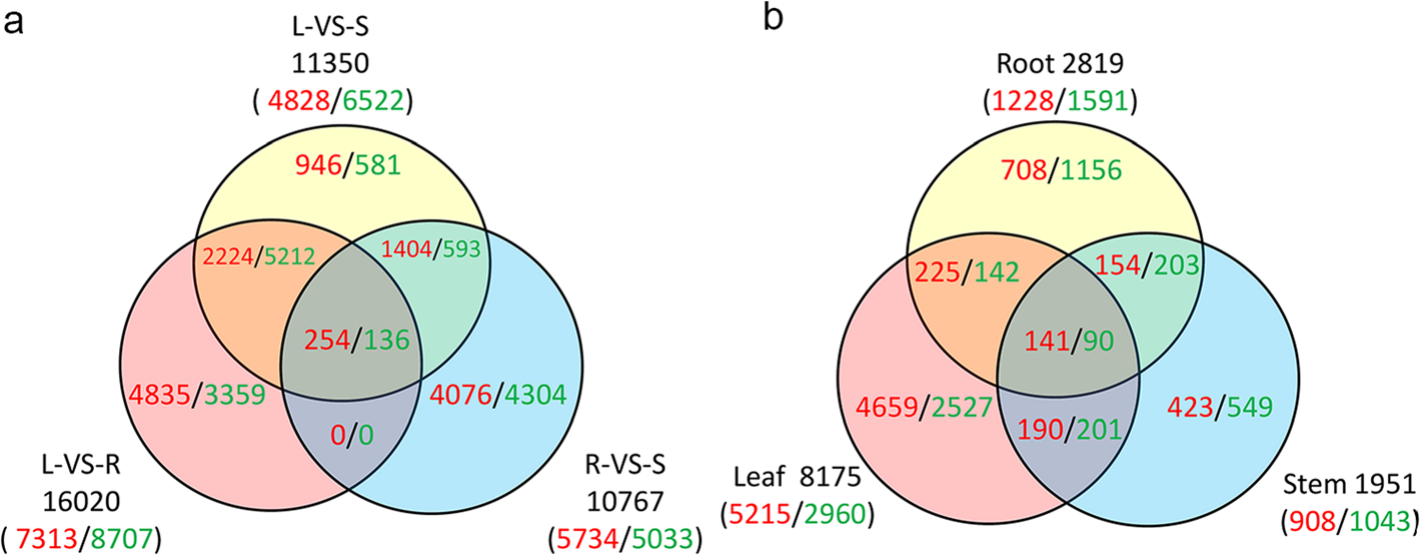
Venn diagrams of DEGs in the roots, stems and leaves of *Populus simonii× P.nigra.***a** The number of DEGs in L-VS-S, L-VS-R and R-VS-S comparisons; **b** The number of salt-responsive DEGs across the three tissues. The numbers in red and green denote up- and down-regulated DEGs, respectively. The numbers in black denote the sum of up- and down-regulated DEGs. R, S and L indicate roots, stems and leaves, respectively

A total of 11,350 DEGs (4828 high expression /6522 low expression), 16,020 (7313/8707), and 10,767 (5734/5033) were identified from a profile of 73,013 polar genes in the comparison groups between leaves and stems, leaves and roots, roots and stems, respectively. There were more highly-expressed DEGs in the roots, compared to the stems and leaves. Among the DEGs, a total of 390 (254/136) DEGs were shared in the all three comparison groups (Fig. 1a, Supplemental Excel 1). These 390 DEGs were mainly involved in different biological processes including plant development, stress responses, metabolism process, hormone signaling etc.. Among the 254 up-regulated DEGs, there were 23 transcription factors, 3 transcription repressors, 14 transporter genes, and a few stress responsive genes such as A/B Barrel Domain (*Dabb*) gene. And there were 12 transcription factors, 7 transporter genes, and also a few stress responsive genes such as early-responsive dehydration stress *ERD3* gene among the 136 down-regulated genes (Supplemental Excel 1).

**Fig. 2:**
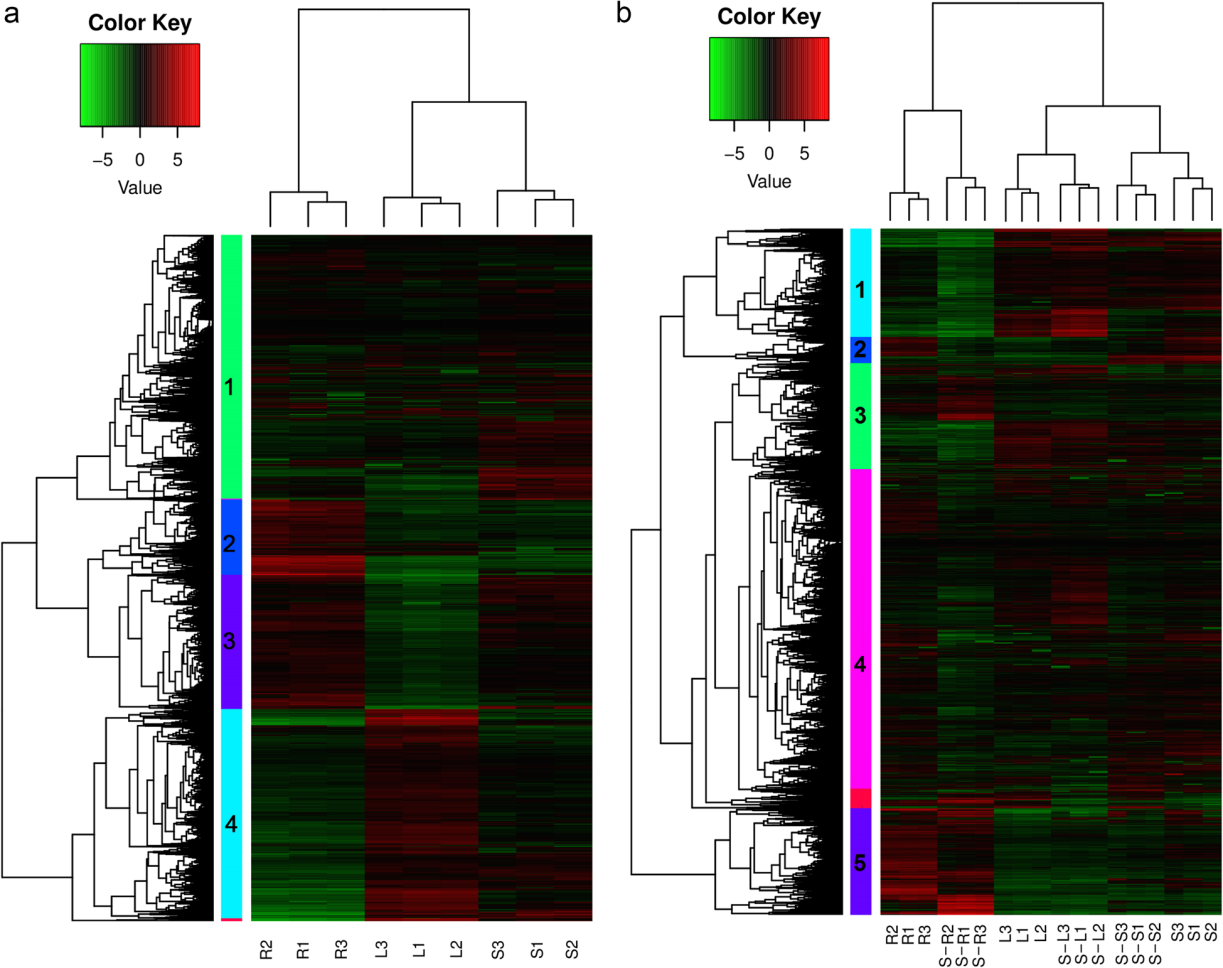
Heatmaps of DEGs in the roots, stems and leaves of *Populus simonii× P.nigra.***a** The heatmap of DEGs in the three tissues of *Populus simonii× P.nigra*; **b** The heatmap of DEGs in the three tissues of *Populus simonii× P.nigra* with salt treatment. Red and green colors indicate up- and down-regulated expression, respectively. The colorful vertical bars denote different gene clusters. R, S, L indicate the roots, stems and leaves under control condition, respectively; S-R, S-S, S-L indicate the roots, stems and leaves under salt stress condition, respectively

Clustering analysis was utilized to identify expression pattern of the DEGs in the three tissues (Fig. 2a). The DEGs can be vertically clustered into 3 groups according to different tissues and horizontally classified into 4 clusters based on mRNA abundances. The cluster 2 displayed high expression in the roots, compared to the leaves and stems; the cluster 3 displayed low expression in the leaves, in comparison with the stems and roots; the cluster 4 was highly expressed in the leaves, while lowly expressed in the roots; the cluster 1 seems to be highly expressed in the stems, compared to the leaves and roots. In addition, the DEGs in the stems and leaves were clustered together, indicating the DEGs shared similar expression pattern in the two tissues (Fig. 2a).

### The salt-responsive DEGs in the three tissues of *Populus simonii×P. nigra*

A total of 2819, 1951, and 8175 DEGs including 1228, 908 and 5215 up-regulated genes (URGs) and 1591, 1043 and 2960 down-regulated genes (DRGs) were identified in the roots, stems, and leaves under salt stress, respectively (Fig. 1b, Supplemental Excel 2). Among them, a total of 231 DEGs (141 URGs and 90 DRGs) were shared across the three tissues. These 231 DEGs were mainly associated with stress responses, such as the auxin responsive *GH3* gene, ethylene-responsive transcription factor *15*, desiccation-responsive gene *29B*, *Dabb* (stress responsive A/B barrel domain) gene, etc.. Among the 141 URGs, there were 9 transcription factors, 4 regulator genes, and a few stress-responsive genes. And there were 8 transporter genes, 3 interacting genes, and a few synthase or enzyme genes among the 90 DRGs (Supplemental Excel 2).

The DEGs can be vertically clustered into 3 groups including 6 subgroups, and the genes in the stems and leaves were clustered together with salt treatment. Horizontally, the DEGs were mainly classified into 5 categories. There were more URGs detected in the leaves than DRGs, while the opposite phenomenon happened in the roots and stems. The cluster 1 was expressed in the roots at a lower level than that in the leaves and stems. The cluster 5 displayed higher expression in the roots, compared to the leaves and stems. The cluster 2 and 4 was down-regulated in the roots under salt stress, compared to control condition. And the other clusters were mixed with URGs and DRGs with salt treatment (Fig. 2b).

### The differentially expressed NACs in the comparisons among the three tissues of *Populus simonii×P. nigra*

**Fig. 3:**
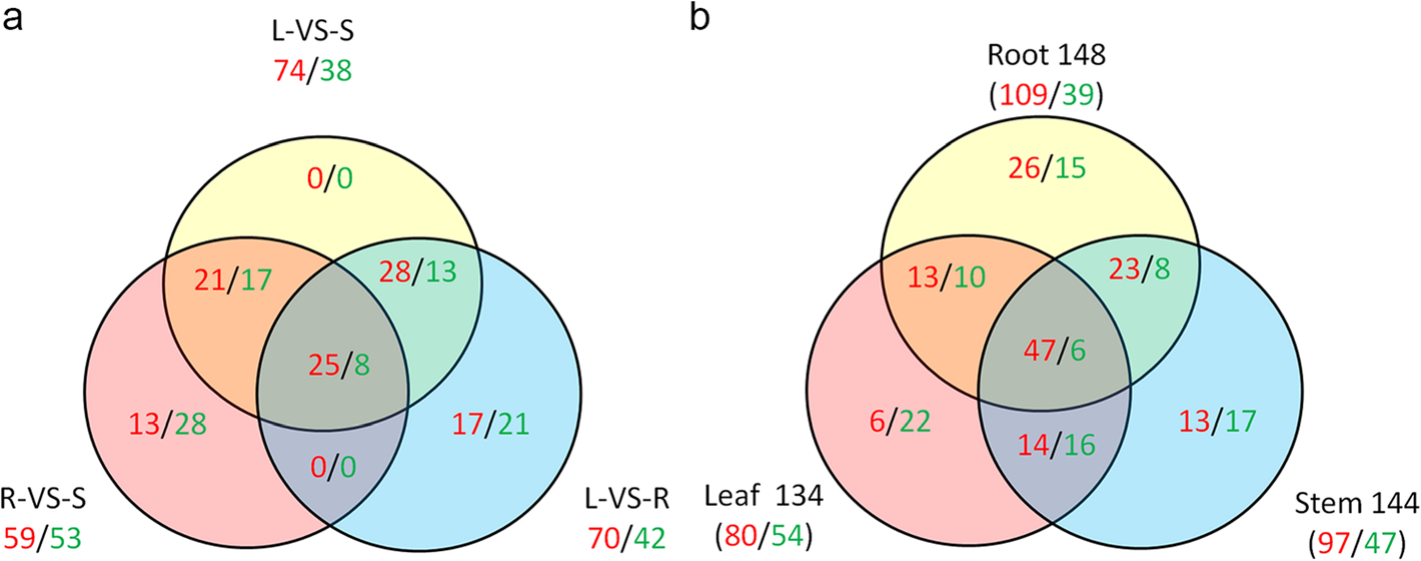
Venn diagrams of NACs in the roots, stems and leaves of *Populus simonii× P.nigra.***a** The number of NACs in L-VS-S, L-VS-R and R-VS-S pair comparisons; **b** The number of salt-responsive NACs across the three tissues. The numbers in red and green denote highly- and lowly-expressed NACs, respectively. The numbers in black denote the sum of highly- and lowly-expressed NACs. R, S and L indicate roots, stems and leaves, respectively

To assess expression pattern of NAC family in different tissues, the mRNA abundances of all 289 NAC members (Supplemental Excel 3) were derived from transcriptome data of the three tissues of *Populus simonii×P.nigra*. Based on FPKM, 227 NACs were detected and 112 out of them were expressed in at least one tissue with FPKM≥4 (Supplemental Excel 3). More highly-expressed NACs were detected in the stems, compared to the roots and leaves, indicating more NACs prefer to specific express in the stem. And respective 74/38, 70/42, 59/53 NACs were identified to be highly/lowly-expressed in the comparative analysis of leaves VS stems, leaves VS roots, roots VS stems (Fig. 3a). As many as 38 NACs (17 high expression/21 low expression) were specifically expressed in the comparison of leaves and roots. And 41 NACs including 13 highly-expressed genes and 28 lowly-expressed genes were specifically expressed in the roots-VS-stems comparison. There was no gene specifically expressed in the comparison of leaves and stems, indicating NACs expressed with a similar pattern in the two tissues. A total of 33 NACs including 25 highly- and 8 lowly-expressed genes were shared in the three comparisons (Fig. 3a). There were 47 (31/16), 43 (31/12), 35 (20/15) NACs whose FC was 2 times higher in the comparisons of leaves and stems, leaves and roots, roots and stems, respectively (Supplemental Excel 4).

**Fig. 4:**
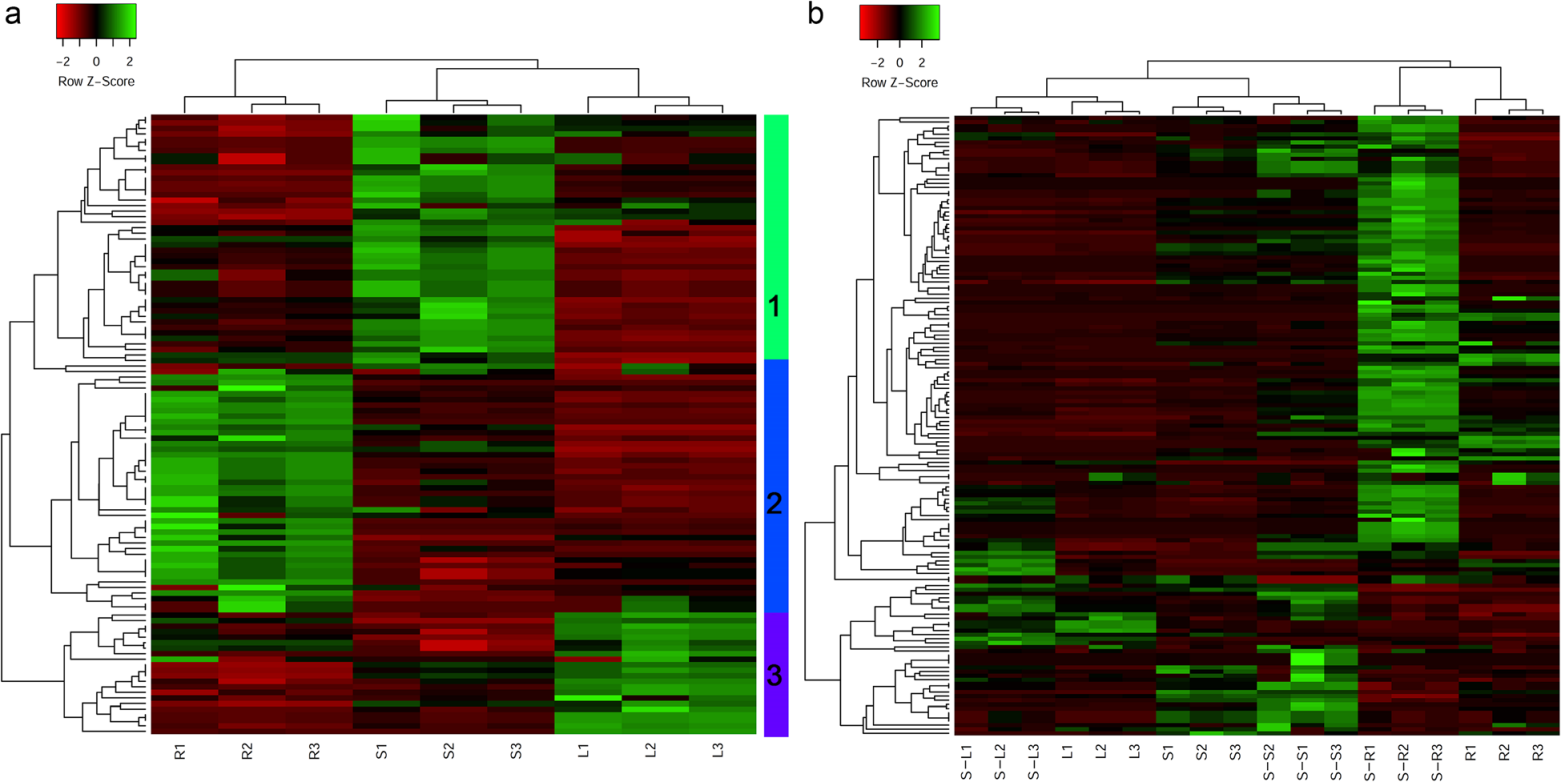
Heatmaps of NACs in the roots, stems and leaves of *Populus simonii×P.nigra.***a** The heatmap of 151 NACs in the three tissues of *Populus simonii×P.nigra*; **b** The heatmap of 112 NACs in the three tissues of *Populus simonii×P.nigra* with salt treatment. Red and green colors indicate low and high expression, respectively. The colorful vertical bars denote different gene clusters. R, S, L indicate the roots, stems and leaves under control condition, respectively; S-R, S-S, S-L indicate the roots, stems and leaves under salt stress condition, respectively

Hierarchical clustering was conducted to identify expression pattern of the 112 NACs in the roots, stems and leaves. The NACs can be vertically clustered into 3 groups. The genes in the leaves and stems were classified into same group, suggesting expression pattern of NACs were similar in the two tissues, which was same to expression pattern of DEGs. Horizontally, the genes can be classified to three clusters. The cluster 1, 2, and 3 displayed high expression in the stems, roots, and leaves, respectively (Fig. 4a). There were more NACs highly expressed in the stems, compared to the roots and leaves, which was different with expression pattern of DEGs in the three tissues.

### The salt-responsive NACs in the three tissues of *Populus simonii×P. nigra*

To facilitate understanding expression pattern of NAC family under salt stress, the mRNA abundances of all 289 NAC members were derived from transcriptome data of *Populus simonii×P.nigra* under control and salt stress conditions. Based on FPKM, there were 258 NACs detected and 151 NACs expressed in at least one tissue with FPKM≥4 (Supplemental Excel 3, Fig. 3b). Out of the 151 NACs, there were 109/39, 97/47, 80/54 genes found to be up/down-regulated in roots, stems, and leaves, respectively (Fig. 3b). The ratio of up-regulated NACs to downed-regulated was 2.79, 2.06, and 1.48 in the three tissues, respectively. There were 41 (26 up/15 down), 30 (13/17), 28 (6/22) NACs specifically expressed in the three tissues, respectively. The numbers of salt-responsive NACs shared by two tissues were 31 (23/8), 30 (14/16), 23 (13/10), respectively. A total of 53 NACs including 47 up-regulated and 6 down-regulated genes were found in common across the three tissues (Fig. 3b). The numbers of salt-responsive NACs whose FC was 2 times higher were 75 (66/9), 32 (26/6), 39 (25/14) in the roots, stems, and leaves, respectively (Supplemental Excel 4).

The clustering analysis of the 151 NACs was performed to investigate the expression pattern of NACs in the different tissues under salt stress. The genes can be vertically classified to 3 groups including 6 sub-groups, and many clusters horizontally (Fig. 4b). Under salt stress, there were more up-regulated NACs in the roots than those in the stems and leaves. The genes in the leaves and stems were classified into same group, which was same to DEGs in the three tissues. The results illustrated salt-responsive NACs and DEGs shared similar expression pattern in the leaves and stems under control and salt stress conditions.

### Expression analysis of 170 non-redundant NACs in the leaves of *Populus simonii×P. nigra* under salt stress

**Fig. 5:**
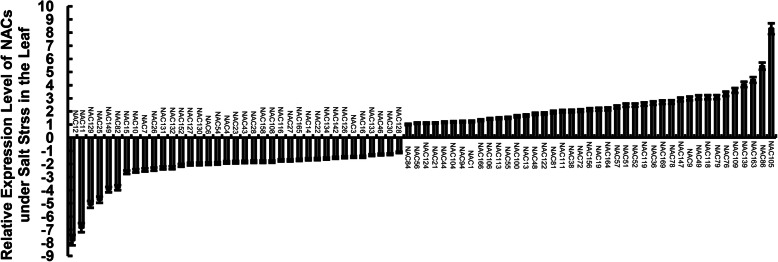
Relative expression level of 170 non-redundant NACs in the leaves of *Populus simonii×P.nigra* with salt treatment. The *Populus simonii×P.nigra* seedlings with new roots and leaves were treated with 150 mM NaCl and water as control, separately. The leaves were harvested at 24 h with three biological repeats. Mean values and deviations were calculated from three independent biological experiments

Based on PlantTFDB (http://planttfdb.cbi.pku.edu.cn/index.php), there are 170 NACs encoding different amino acid sequences with different transcript. The relative expression level of all 170 non-redundant NACs was quantified in the leaves of *Populus simonii×P.nigra* by RT-qPCR. A total of 78 NACs were in response to salt stimulus, including 41 up-regulated genes and 37 down-regulated genes (Fig. 5). There were 86 salt-responsive NACs including 50 up-regulated genes and 36 down-regulated genes (Supplemental Excel 3) based on RNA-Seq data of the 170 NACs. The results indicated the expression of most NACs embody unanimity by RNA-Seq and RT-qPCR.

### Temporal and spatial expression analysis of 6 NACs in *Populus simonii×P. nigra*

**Fig. 6:**
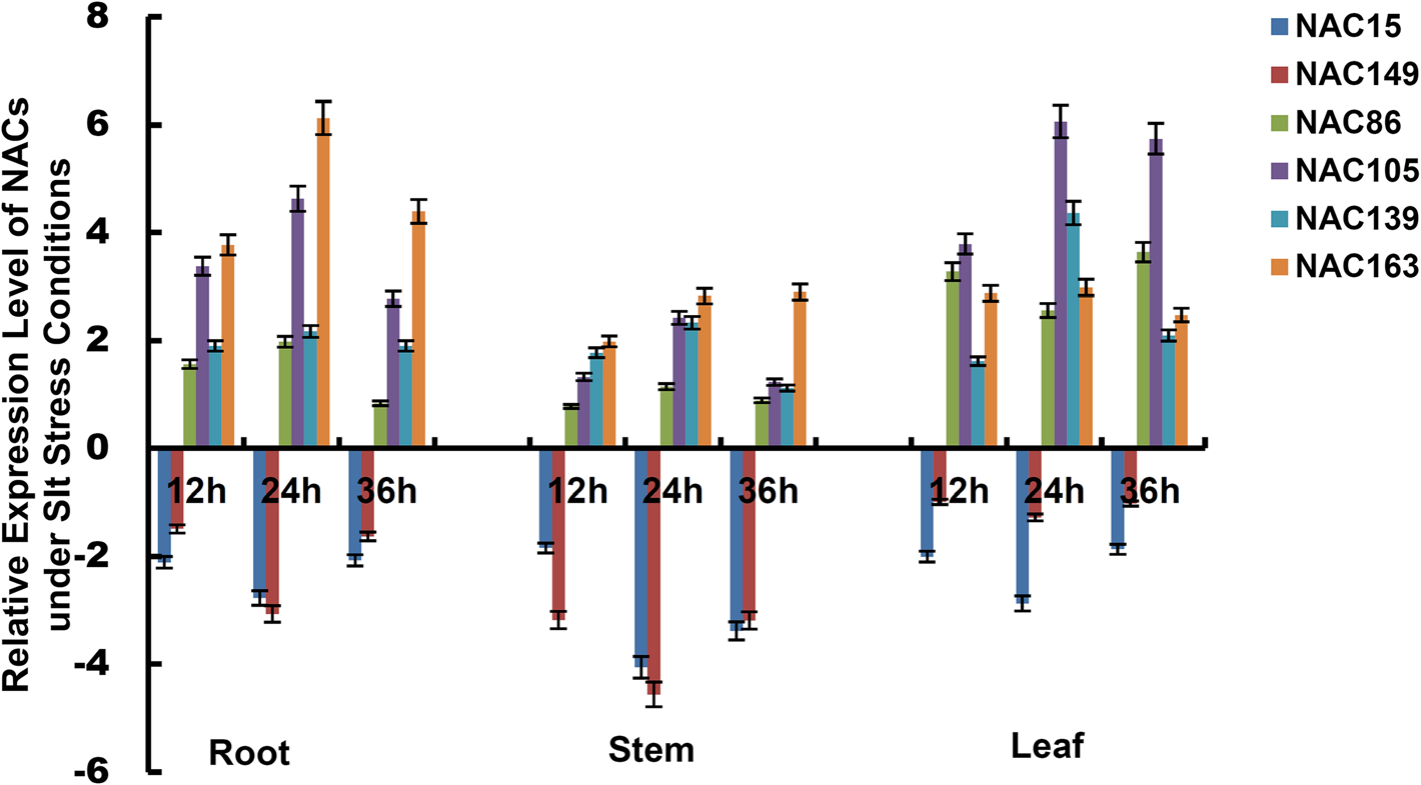
Expression pattern of 6 NACs in the roots, stems and leaves of *Populus simonii×P.nigra* under salt stress. The *Populus simonii×P.nigra* seedlings with new roots and leaves were treated with 150 mM NaCl and water as control, separately. The roots, stems and leaves tissues were harvested at 0, 12, 24 and 36 h with three biological repeats, respectively. Mean values and deviations were calculated from three independent biological experiments

To further examine temporal and spatial expression pattern of NACs, we quantified relative expression level of four randomly up-regulated genes (*NAC86*, *NAC105*, *NAC139* and *NAC163*) and two down-regulated genes (*NAC15* and *NAC149*) in the three tissues with salt treatment for 0, 12, 24 and 36 h, respectively. The results indicated the expression pattern of four up-regulated NACs displayed similar expression pattern in the different tissues across the whole time course. The expression trend increased during 12–24 h, reached peak at 24 h, and then decreased during 24–36 h, which was opposite for two down-regulated genes. The relative expression level of up-regulated NACs was generally higher in the roots and leaves, compared to that in the stems, whereas the expression change of down-regulated genes was higher in the stems, compared to that in the roots and leaves (Fig. 6). The results validated a few NACs showed different temporal and spatial expression patterns in *Populus simonii*×*P.nigra*.

### Phylogenetic analysis of NACs from *Populus* and *Arabidopsis*

**Fig. 7:**
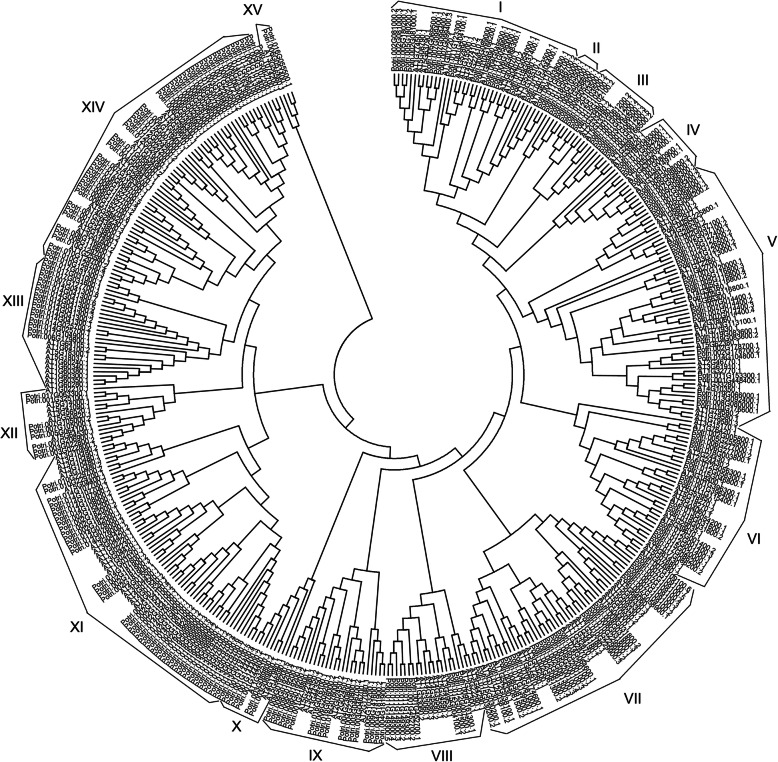
Phylogenetic tree of 289 NACs from Populus trichocarpa and 138 NACs from Arabidopsis thaliana

To identify homologous relationship of NACs from *Populus* and *Arabidopsis*, a phylogenetic tree containing 289 NACs from *Populus trichocarpa* and 138 from *Arabidopsis thaliana* was constructed with their deduced protein sequences (Fig. 7). The NAC proteins were clustered into 15 distinct clades (I to XV). The largest subgroup was XI with 61 NACs including 43 from *Populus* and 18 from *Arabidopsis*, and the smallest subgroup was XV with 4 members (Fig. 7). Noticeably, all other subgroups contained NACs from both *Populus* and *Arabidopsis* except subgroup II with only 5 poplar NACs, indicating the subgroup may be acquired from their divergence with common ancestor.

In the study, we identified a total of 112 NACs differentially expressed in the comparisons of leaves and stems, leaves and roots, roots and stems of *Populus simonii×P. nigra*, which were mainly clustered into subgroups I, V, VI, IX, VII, XI, XIII, XIV. Based on homologous analysis of the NACs from *Arabidopsis* and *Populus*, 57 out of the 112 NACs may be associated with plant development processes, which were mainly clustered in the V, IX and XI subgroups (Supplemental Table 2, Fig. 7). And 18 out of the 57 NACs were found to participate in xylem formation or secondary wall synthesis according to gene annotation (Supplemental Excel 3). On the other hand, a total of 53 salt-responsive NACs were identified to be shared across the three tissues of *Populus simonii×P. nigra*, which were mainly clustered into I, VI, VII, IX and XI subgroups. As well, 20 out of the 53 NACs were deduced to be involved in stress responses according to homologous relationship of the NACs between *Arabidopsis* and *Populus*, and the most of 20 NACs were clustered into the subgroups I and VII (Supplemental Excel 3, Fig. 7).

## Discussion

Given previous studies in recent years, biological function of poplar NACs has been highlighted in wood formation and stress responses. NACs act as master switch in transcriptional control of secondary cell wall biosynthesis by regulating a suite of downstream TFs and wood biosynthetic genes in *Populus trichocarpa* [39]. A total of 12 NACs from *Populus trichocarpa* were identified to be expressed in xylem tissue and phloem fiber, and overexpression of the genes induced ectopic secondary wall thickening in poplar leaves [40]. As well, many poplar NACs were identified to be stress-responsive and overexpression of the genes contributed to stress tolerance of transgenic plants. For instance, a total of 76 NACs were screened to be salt-responsive in the leaves of *Populus alba×Populus glandulosa* by RNA-Seq and overexpression of *NAC57* enhanced salt tolerance in transgenic *Arabidopsis* [41]. Three NAC transcription factors of *Populus euphratica* were proved to differentially regulate salt and drought tolerance in transgenic *Arabidopsis* [42]. In this study, the potential functions of whole NAC family, especially in wood formation and stress responses, were explored in *Populus simonii×P. nigra*, which provides a very useful reference for functional analysis of poplar NACs.

With technology improvement of NGS and bioinformatics, RNA-Seq approach combined with phylogenetic analysis has been exploited as a promising technology for preliminary identification of tissue-specific and stress-responsive NACs in many plant species. For example, there were 152 full-length NACs detected in soybean genome and 38 newly predicted stress-related *GmNACs* were identified based on transcriptome analysis and phylogenetic analysis [35]; 136 NACs were identified by comprehensive genome-wide analysis in potato and several *StNACs* were predicted to be tissue-specific, stress- and hormone responsive by RNA-seq and comparative phylogenetic analysis with *Arabidopsis* [36]. In the study, the mRNA abundances of all 289 poplar NACs were profiled in the roots, stems and leaves of *Populus simonii×P. nigra* by RNA-Seq. A total of 112 NACs were screened to be differentially expressed among the comparisons of the three tissues. As many as 148, 144, 134 NACs were detected to be salt responsive in the three tissues, respectively. More importantly, 18 potential poplar NACs associated with wood formation and 20 involved in stress responses were identified from RNA-Seq data by comparative phylogenetic analysis with *Arabidopsis*. In particular, several NACs among them have been confirmed to fulfil key roles in wood formation and stress responses. The study provides a more comprehensive and efficient access for function identification of NAC family through the correlation of transcriptome screening and phylogenic analysis in the different tissues of *Populus*.

Segmental duplications contribute significantly to the expansion of NAC family in *Populus* [8]. There are a total of 289 putative NACs in *Populus trichocarpa* and 119 out of them retain segmental duplicates with other genes [2]. However, the NACs displayed a high divergence rate of expression patterns after segmental duplications and the majority of NACs showed specific temporal and spatial expression patterns in *Populus trichocarpa* [8]. In the study, the expression of 6 randomly selected NACs including four up-regulated genes and two down-regulated genes showed specific temporal and spatial patterns in the three tissues of *Populus simonii×P.nigra* under salt stress. Moreover, 57 NACs were identified to be differentially expressed in the three tissues of *Populus simonii×P. nigra* and perhaps associated with plant development processes based on homologous relationship of the NACs from *Arabidopsis* and *Populus* (Supplemental Table 2, Fig. 7). Among the 57 NACs, 18 genes were perhaps involved in wood formation (Supplemental Excel 3). In details, 8 NACs are homologous with *VNI2*, which negatively regulates xylem vessel formation in *Arabidopsis* [43]; 4 NACs are homologous genes of *Arabidopsis SND2*, which regulates expression of the genes involved in secondary cell wall development [44]; 3 NACs hold homology relationship with *XND1*, which negatively regulates lignocelluloses synthesis in *Arabidopsis* [45]; 3 NACs are homologous with *NST1/3* and *VND1*, respectively. *VND1* contributes to cotyledon xylem vessel formation and *NST1/3* are key regulates of secondary wall formation [46, 47]. And other 39 NACs are associated with other plant developmental processes such as leaf senescence, root cap development, embryogenesis, cell proliferation, shoot apical meristem formation, flowering time etc.. Particularly, our previous study has stated one poplar NAC gene, *NAC15*, enhanced wood formation by regulating lignin- and cellulose-related genes in transgenic tobacco [48].

It is a well-known approach for function analysis of NAC family genes through the correlation of function and phylogeny in plants. A total of 53 salt-responsive NACs were identified to be shared in the roots, stems and leaves of *Populus simonii×P. nigra* in the study. And 20 of the 53 NACs were probably involved in stress responses according to homologous analysis of the NACs from *Arabidopsis* and *Populus* (Supplemental Table 2, Fig. 7). Among the 20 NACs (Supplemental Excel 3), 9 genes hold homology relationship with *ATAF1* gene from *Arabidopsis*, which had dual function in abiotic and biotic stress responses [27]; 4 NACs are homologous with *AtNTL7*, which contributed to ER stress resistance in *Arabidopsis* [49]; 2 NACs are homologous genes of *JUB1*, which enhanced tolerance to drought, heat and salinity in transgenic *Arabidopsis* [50]; 2 NACs are homologous genes of *NTL9* from *Arabidopsis*, which was involved in cross-talk between leaf senescence and osmotic stress responses [51]; 1 NAC is homologous with *ANAC078*, which regulated flavonoid biosynthesis under high light stress [52]; 2 NACs, *NAC13* and *NAC105*, are homologous with *Arabidopsis RD26*, which functioned as a transcriptional activator under abiotic stress [53]. In particular, our previous study has confirmed overexpression of *NAC13* enhanced salt tolerance significantly in transgenic *Populus alba×Populus glandulosa*. And *NAC13* antisense mRNA transgenic lines showed significant decrease in salt tolerance compared to wild poplar [54]. Also we have proved *NAC105* was the most significant up-regulated gene among the whole NAC family in the leaves with salt treatment in the study, and it showed regulatory function in salt stress tolerance in transgenic plants (unpublished data).

## Conclusions

In the study, a total of 112 NACs (38.8%) were identified to be differentially expressed in the comparisons of leaves and stems, leaves and roots, roots and stems of *Populus simonii×P. nigra* by RNA-Seq. Under salt stress, as many as 109/39, 97/47, 80/54 NACs were detected to be up/down-regulated in roots, stems, and leaves, respectively. Among them, a total of 53 salt-responsive NACs were identified to be shared across the three tissues. RT-qPCR results indicated 78 NACs including 41 up-regulated and 37 down-regulated genes were identified to be salt-responsive among 170 non-redundant NACs, which was similar to RNA-Seq results. The expression pattern of 6 NACs indicated a few NACs showed specific temporal and spatial expression patterns in the three tissues of *Populus simonii×P.nigra* under salt stress. A total of 18 potential NACs associated with wood formation and 20 involved in stress responses were identified by transcriptome screening and phylogenic analysis of differentially expressed NACs in different tissues under salt stress. The study provides a very useful reference for functional characterization of NACs in poplar.

## Methods

### Plant materials

The growing twigs of *Populus simonii×P. nigra* can sprout new roots and leaves by hydroponic culture at room temperature with 16/8-h light/dark cycles and 70% relative humidity [38, 48, 55]. A total of 42 twig seedlings from one clone of wild-type *Populus simonii×P. nigra* in experimental forest of Northeast Forestry University were obtained as plant materials [48]. Among them, 21 twig seedlings with new roots and leaves at about one-month-old were treated with 150 mM NaCl, and the other 21 were cultured in water as control. The roots, stems and leaves from 24 seedlings were harvested with three biological replicates (one seedling as one biological replicate) under control and salt stress conditions at 0, 12, 24, 36 h, respectively, and frozen in liquid nitrogen for RT-qPCR. The respective roots, stems and leaves from 18 seedlings under control and salt stress conditions for 24 h were collected with three biological replicates (three seedlings as one biological replicate) for RNA-Seq. Three biological replicates were prepared for each treatment at each time point.

### Gene expression analysis using RNA-Seq

Above 18 samples were sent to GENEWIZ Company for RNA-Seq with Illumina Hi-seq2000 platform. The raw RNA-Seq data was processed as described in our previous studies [38, 55]. The mRNA abundance of genes was quantified as FPKM (fragment per kilo bases per million reads) [48].

The DEGs were identified following the criteria: false discovery rate (FDR) ≤0.05 and Log_2_ (fold change, FC) ≥2. Hierarchical clustering of DEGs was conducted by Gene Cluster 3.0 and visualized by Java Treeview [56]. The NACs with FPKM≥4 in at least one tissue under control or stress condition were applied to count up- or down-regulated expression by FC [55]. Venn diagrams of DEGs and NACs were constructed by VENNY 2.1 software (http://bioinfogp.cnb.csic.es/tools/venny/index.html).

### Gene expression analysis using RT-qPCR

The primer pairs of 170 non-redundant NACs and actin gene as internal control (Supplemental Table 1) were designed based on Phytozome12 database (https://phytozome.jgi.doe.gov/pz/portal.html). Experimental operating system of RT-qPCR was referred to our previous studies [38, 48].

### Phylogenetic analysis of NACs from *Populus* and *Arabidopsis*

The information of NAC family members from *Populus trichocarpa* and *Arabidopsis thaliana* was derived from PlantTFDB (http://planttfdb.cbi.pku.edu.cn/) (Supplemental Table 2). Phylogenetic tree including 289 NACs from *Populus trichocarpa* and 138 NACs from *Arabidopsis thaliana* was constructed by MEGA X with Neighbor-Joining method [57].

## Supplementary information

**Additional file 1: Table S1.** List of primer pairs of actin gene and 170 non-redundant NACs from *Populus trichocarpa. (DOCX 26 kb)*

**Additional file 2: Table S2.** Homologous information of NACs from *Arabidopsis thaliana* and *Populus simonii×P.nigra. (DOCX 27 kb)*

**Additional file 3: Excel 1** DEGs in the roots, stems and leaves of *Populus simonii×P.nigra. (XLSX 2947 kb)*

**Additional file 4: Excel 2** Salt-responsive DEGs in the roots, stems and leaves of *Populus simonii×P.nigra. (XLSX 1212 kb)*

**Additional file 5: Excel 3** List of related NACs including all 289 NAC members, 112 NACs expressed in at least one tissue with FPKM≥4 among the three tissues, 151 NACs expressed in at least one tissue with FPKM≥4 with salt treatment, 86 salt-responsive NACs among 170 non-redundant NACs, 18 potential NACs associated with wood formation and 20 potential NACs involved in stress responses.

**Additional file 6: Excel 4** List of differentially expressed NACs with FC > 2 in the comparisons of three tissues or in each tissue with salt treatment.

## Data Availability

The data generated during this study are included in this published article and its supplementary information files. The raw sequencing data used during the study have been deposited in NCBI SRA with the accession number SRP267437.

## Abbreviations

*TF*: Transcription factor
*NGS*: Next generation sequencing
*DEGs*: differentially expressed genes
*NACs*: NAC genes
*URGs*: up-regulated genes
*DRGs*: down-regulated genes
*FC*: fold change
*FPKM*: Fragment per kilo bases per million reads
*FDR*: false discovery rate
*VND*: vascular-related NAC domain
*SND*: secondary wall-associated NAC domain
*NST*: NAC secondary wall thickening
