# Supplementary material for: Transcriptome analysis of salt-responsive and wood-associated NACs in Populus simonii × Populus nigra
Source: BMC Plant Biol. 2020 Jul 6;20:317. doi: 10.1186/s12870-020-02507-z (PMC7336439; doi:10.1186/s12870-020-02507-z)
Supplement: Supplementary file 1 — Additional file 1: Table S1. List of primer pairs of actin gene and 170 non-redundant NACs from Populus trichocarpa. (DOCX 26 kb) [file 12870_2020_2507_MOESM1_ESM.docx]

Supplemental Table 1 List of primer pairs of actin gene and 170 NAC genes from *Populus*

| Name | ID | Forward primers | Reverse primers |
| --- | --- | --- | --- |
| Actin | JM986590 | ACCCTCCAATCCAGACACTG | TTGCTGACCGTATGAGCAAG |
| NAC1 | Potri.001G061200.1 | CTCTGGCTACTGGAAGGCAACT | TTGCAGGCTGTGGTTTCAGTGC |
| NAC2 | Potri.001G080900.1 | GGGAGTGGTACTTCTTCTGTAG | CACAAGCAGGAAGGCGAAATTC |
| NAC3 | Potri.001G120000.1 | CTCAACCTTACGCACCATACCA | TGACTGCAAGGTGTTCCCATTG |
| NAC4 | Potri.001G144400.1 | CAGTAGCTCGAGCAATGTGGAT | AACTCTCCATTGAAGCAGCTGG |
| NAC5 | Potri.001G206900.1 | GATCCATGGCAACTTGATGGCA | GCTATCACTTGCAAGCACAAGC |
| NAC6 | Potri.001G218800.1 | CGACATCGAACCATGGGATCTT | AACATAACCAGGAGAGCCTGTG |
| NAC7 | Potri.001G220500.1 | CGGCAATGATCAAGGTGCTCAA | ATCTCCTTGCCACACACAGTAG |
| NAC8 | Potri.001G256600.1 | GACTGCGTTCTGTGCAGGATTT | AGCTAGCCTCTATAGCAGAGAG |
| NAC9 | Potri.001G325100.1 | GAAGCGAAAGGTCCTTGGTCTT | CCAGAAGGTATATAACCAGCGG |
| NAC10 | Potri.001G343800.1 | CAAAGCTACCTGGTGCCAAGAA | GAACTCCATTGTCCATCACAGG |
| NAC11 | Potri.001G396300.1 | GTAGGGCCATTGCTGAAGTTGA | CAATAACCAGCTTCAGTGGCTC |
| NAC12 | Potri.001G396400.1 | GTAGGGCCATTGCTGAAGTTGA | GTGCACCAGTCTTTGAGCTATG |
| NAC13 | Potri.001G404100.1 | GCTCTTACCAAGCAAGGCGATA | AGGCGGTATTCATGCATGATCC |
| NAC14 | Potri.001G404400.1 | GCAGCAACTTCAGGGTATTGGA | TTTCCCAAGTCACATCCTGGTG |
| NAC15 | Potri.001G448400.1 | CAGGGACTAGAACAAATCGAGC | TGTCGTTGGTGCTGTCATCAAG |
| NAC16 | Potri.001G452700.1 | CGTACGAACTGGATCATGCATG | TATCAGGAGAGGACTTAGCAGC |
| NAC17 | Potri.002G005800.1 | GCCACAGGCAAAGATAGAGAAG | ATCACCCATTCCTCCTTACACG |
| NAC18 | Potri.002G037100.1 | GACTTGCATACTTGCGAGCCAT | ACAGTACGATCCTTCCCTGTAG |
| NAC19 | Potri.002G057200.1 | GTACGATCCATGGGATCTTCCA | TTGAGGCCAATGCACTCATGTG |
| NAC20 | Potri.002G061300.1 | CACCTAACGGTGATCGAACTGA | CATCATCAGCCCAGTCTTCTTC |
| NAC21 | Potri.002G081000.1 | CTCTACAAGTTTGACCCATGGG | CTTGATTCCAACTGTCTTCGGC |
| NAC22 | Potri.002G154000.1 | CTTGCTACTCATGGCAGAGGTT | TTCCTGGCAGGTTGATTTCCAG |
| NAC23 | Potri.002G154100.1 | GGATACTGGAAGTTGACCTGCA | AGAGAGGCTAGAATTGGTGACG |
| NAC24 | Potri.002G154200.1 | GGCTATGATCACGTAGTCAGCA | CCTGTTAGCCCTCTGACTTTTG |
| NAC25 | Potri.002G178700.1 | GTGACAAGGTGATATACGGCAC | TGGAGACATTGGTTTCACTGGG |
| NAC26 | Potri.002G181900.1 | GGCTAAACGCACAAATGAGGTG | ATAGGCTCGCCTTCATCATCAG |
| NAC27 | Potri.002G182000.1 | CGGGCTAATAGGACAACTGAAG | TGGATGGTACTCGTGTATGACC |
| NAC28 | Potri.002G182300.1 | CCCTGGGCTTTCTACATATCAG | CGGTCTTGACTCCATTAGGAAC |
| NAC29 | Potri.002G182400.1 | GCTAAGCGTAACAAGGAGGTGA | GCTTCAAAACCCGAGGTAGAAG |
| NAC30 | Potri.003G022800.1 | CAGATGTCATCCCGGATCTTGA | TAGGTTCTTCAACGTGGCCAAC |
| NAC31 | Potri.003G046700.1 | CCGGATGATCCGAACTGAGAAT | GGATGATCTTCTACTCCAGCTC |
| NAC32 | Potri.003G089800.1 | GAGACAGGACAGACTGGGTAAT | TGAGATGTTCGAAGGCTCGTTC |
| NAC33 | Potri.003G103500.1 | GAGGCTGGATGACTGGGTTTTA | CAGGCAGATCAGTCCTATCTTC |
| NAC34 | Potri.003G113000.1 | CCTACCGGAACTCGCACAAATA | ACAACCCATCCTTTTGCCTGAG |
| NAC35 | Potri.003G149400.1 | CCCCTTCCTTGCGATTCAATCA | TTTGGGTTCCTTTCCACGTACC |
| NAC36 | Potri.003G149700.1 | CGAGCCATGGGATCTTCCAAAA | TCAGGCCAATGCAGTAATGGAG |
| NAC37 | Potri.003G166500.1 | GTGATTTGGAGCAGGAGAGGTA | TCCAATCAGTCCTAGTGCCATG |
| NAC38 | Potri.004G038000.1 | GCCTAACAGAGCTGCAACTTCA | TTGTTGGCAGAATCAGCAGCAG |
| NAC39 | Potri.004G049300.1 | GAGTGAAGTTTGATCCGACGGA | CTTACTCCTGGTAGCTTCTCAG |
| NAC40 | Potri.004G049500.1 | CAGCAGGATCAGGTTACTGGAA | GGTATCATCAAGAAGCCGGTAC |
| NAC41 | Potri.004G081000.1 | GCAGCATAGGTTCACATTGCCT | TCAGCAGTGGCATAGTTTCCAC |
| NAC42 | Potri.004G107200.1 | GACGAACATTGCACTGTCACAG | CATCATCAACACCGTCAGCTTG |
| NAC43 | Potri.004G107400.1 | CCACTGATCAAGAACTGGTCCA | ATTTTGCGGGTGACATGGGAAG |
| NAC44 | Potri.004G119400.1 | GGTGAACAACTTCCTGCTAACC | ACCGTCAGGATCGATCTCATAG |
| NAC45 | Potri.004G181900.1 | GGCTACCTCAACATCTATCGTC | CATTCTCTTCGGGTCCGATAAG |
| NAC46 | Potri.004G230800.1 | GGATGATCCGAGCTGAGAATTC | TATCCCAGCTCTCTTGTAGACG |
| NAC47 | Potri.005G058900.1 | CTCATATGTGCCAGAGAGCACA | GAGCTAGAGCTTGCTAAGATGG |
| NAC48 | Potri.005G064100.1 | CTCTGTGGAGTGAAAACCAGTG | TTCTACACCTTCGGGACCAATG |
| NAC49 | Potri.005G069500.1 | GATCCTTGGCAGCTTCCAGAAA | ACCAATGGGTTTATCTGCTCCG |
| NAC50 | Potri.005G082700.1 | GACATCTACGCCTATCAACCTC | CAATGCTCGTCAATAGCGACAC |
| NAC51 | Potri.005G098000.1 | CCCTTGGGATCTACCAAAGTCA | TCTTGAGTCCAATGCTGTCACG |
| NAC52 | Potri.005G098200.1 | CTAATCGTGCAACAGCTTCTGG | AAGAGGTCCTTCAAGGCGAAAC |
| NAC53 | Potri.005G103200.1 | CTTGGGACTTGCCAGGATTATC | CATGACCCAATCCGTTTTGCTC |
| NAC54 | Potri.005G116800.1 | GTCAATGGAGTCTTGTGTCCCA | CTGCTCTTCATATCCGATCCAG |
| NAC55 | Potri.005G180200.1 | CTCTACAAGTTTGACCCATGGG | TCTTGATCCCAGCTGTCTTTGG |
| NAC56 | Potri.005G200100.1 | GAGTTGCCTGGGCAATCGATAT | CAATCAGTTCGTTGACCACTCG |
| NAC57 | Potri.005G205400.1 | GGGATCTTCCAATTAGCACTGC | GCATTTCTTGAGACCGATGCAC |
| NAC58 | Potri.005G225800.1 | GCTAAGCTCAACGCAACTGAGT | GATTCCATTGGGAGCTCTGTTC |
| NAC59 | Potri.005G255900.1 | CAGTGCTTCCACTGGTGCTTTA | CCTGCAAATCACCCATTCATCC |
| NAC60 | Potri.006G028300.1 | GGGAGGTGATGATGCTCTCTAT | TGAACCATACTCCAGCAGTGTC |
| NAC61 | Potri.006G028700.1 | GCCACAAGGAAGGTGATGATGT | CAACAACCATGCTTGTCTCGTC |
| NAC62 | Potri.006G028900.1 | CAGGTCACAAGGTAGGTGATGA | GTCGTTGGTTGTTCTGGTAACG |
| NAC63 | Potri.006G029200.1 | GGTGACGGTGTTGAAGAAGTGA | GCAAGACAAACCCCTTCTGATG |
| NAC64 | Potri.006G029300.1 | CCAATAGTACTCAGAGGATGCG | TACTCCAGCAGTGTCCAACAAC |
| NAC65 | Potri.006G029600.1 | GACTACAATCTTCACGGTGGAG | TTCACTACATCAACGCTGCCAC |
| NAC66 | Potri.006G030600.1 | GCCGATATGATCAAGCTCTGCA | TCACTTCTTCAACACCGTCACC |
| NAC67 | Potri.006G030800.1 | GACTACAATCTTCACGGTGGAG | CTTTCACTACATCAACGCTGCC |
| NAC68 | Potri.006G051400.1 | CCGTGGAAGCAACAACAATGAC | ATCGCATGGCATTGCTATTGCC |
| NAC69 | Potri.006G129400.1 | GCAGCAGCTTCAGGTTATTGGA | ACACAGCACCCAATCATCCAAC |
| NAC70 | Potri.006G152700.1 | GATTGGTTAGGTCTACCAGCAG | CATCTCTTGTGACTCCTGGAAG |
| NAC71 | Potri.006G179800.1 | GTGCATCGCCATTGTAATGGCA | TTGTGTGGGAGGATCTACAAGC |
| NAC72 | Potri.006G209200.1 | CAGGAGATTTGTGCCATGGAGA | TCGGTTTTTCGTCCTCTTGGAG |
| NAC73 | Potri.006G231300.1 | GCCAGAGAACCTTGGCAAATCT | TTTTTGATGGCTGCGACTTGGC |
| NAC74 | Potri.006G277000.1 | CAACAAGTGCGAACCATGGGAT | CAACTGTGACGTGACACTGTTG |
| NAC75 | Potri.007G014400.1 | GATCTACAAGAGAGATGCCGGA | GGGTTTTCCTCATGCCAATGAG |
| NAC76 | Potri.007G065400.1 | GGAAGGACAGACATATCCTACG | TTTGGCCACAACTTCTCTGGTG |
| NAC77 | Potri.007G066300.1 | CCAAGCAGTGTGGGAGATAATG | TCTTGAGTCCAATGCAGTCACG |
| NAC78 | Potri.007G099400.1 | GTATTGGAAAGCAACCGGAGCA | GTCCTGTCAACATTAGCAAGGC |
| NAC79 | Potri.007G105000.1 | CTGATCTTGGTCTCTATCCCCA | TCTACACCTTCAGCACCAATGC |
| NAC80 | Potri.007G109100.1 | CAAGTGGGGTAAGAAAGAAGCG | GATGAGTACGTCCTCTGTCAGA |
| NAC81 | Potri.007G127700.1 | CCTTGAATCAAGAACCACGGAG | TGGCATTGTTGAATCTGCAGGC |
| NAC82 | Potri.007G135300.1 | GCCAAGACCAGGCAAGAATTCA | CCTGGTAACTTTTCTGGGTGTG |
| NAC83 | Potri.008G031800.1 | CGTGGGATCTTCCAGATAAGTC | CCACAACTTGTGAGTTCTGACG |
| NAC84 | Potri.008G080000.1 | GAGCCATGGGATCTCAGAGATA | ACGTCCAGTGTAGAAGACTAGG |
| NAC85 | Potri.008G081500.1 | GGGACTTGCCAGACAAGTCATT | TCTGAGACTGCACTGGTCTATC |
| NAC86 | Potri.008G089000.1 | CTTCGTAACCAAGCCACTTCCA | TCACTCCATTTGGGTACTTGCG |
| NAC87 | Potri.008G116600.1 | GATGTTGTTCAAGAGTGGCCTG | CCTGGTAGATTCTTAGGATGGG |
| NAC88 | Potri.009G019200.1 | CTGCAACGTGTGATTCCAGAGA | GTTGCTGTAGTCCGATTAGGTC |
| NAC89 | Potri.009G052200.1 | CCAGATTCGCTAATGACCGTGA | ATCTGCAAGATACTCGGGATCG |
| NAC90 | Potri.009G052300.1 | GAGCCTTTCAAGCCATCACCAA | AACCAGCTCATGGTTATGAGGG |
| NAC91 | Potri.009G072100.1 | GACTTCCATTGCCTAACACGGT | CTTGTCACCTTTCCATGTACCC |
| NAC92 | Potri.009G141600.1 | GGCTACCTCAACATCTATCGTC | CCTTTTTGGGTCCGATAAGCT |
| NAC93 | Potri.009G161300.1 | CATCCAATCGGAGAATGAGTGG | CCTACGTGGAACACAAGAGTTC |
| NAC94 | Potri.010G129700.1 | CCAGGTGTTAAGCAAGATGGCA | TTTGCCTCCTCTGACAGTACTC |
| NAC95 | Potri.010G166200.1 | CCTAACAGAGCAACTGTGTCAG | ATGGATCCATTCTGCTTGCTGG |
| NAC96 | Potri.010G174600.1 | GACCAGTGAACTCTCAAAAGCG | TTAAACTTGATGGTGCAGCGCC |
| NAC97 | Potri.010G176600.1 | CTAGTCTTCTACACTGGACGTG | AAGAGAAGAAGCGCTAGCCTTC |
| NAC98 | Potri.010G229700.1 | GGACCTTGCAGATAAGTCAAGG | TTGCAAGGAGTTGACACTCTCG |
| NAC99 | Potri.010G229900.1 | GACCCTTGTGTATCATCAGGGT | ATACTGCTCCCCATTCTTTGGC |
| NAC100 | Potri.011G046700.1 | GAGCTGCAACTTCAGGGTATTG | TTGTTGGCAGAATCACCAGGAG |
| NAC101 | Potri.011G058400.1 | GAGTTCCAAGATCAGGGTGGTA | CCTGGTAGATTCTCAGGATGAG |
| NAC102 | Potri.011G058600.1 | CAAGCCAATCCTCACCTCCAAT | ATCATCCAATCGCATCGATCCC |
| NAC103 | Potri.011G115400.1 | CCATCGCTGAAGTTGACCTCAA | TTCATTCCCACAAGTGCACCAG |
| NAC104 | Potri.011G121300.1 | CTGTCAACCTCTCCAAATCTGG | AACTGAAGCCTTTCTTGAGGGC |
| NAC105 | Potri.011G123300.1 | CACGACAGAGGGACGTAAAGTT | CCGACACAATACCCATTCATCC |
| NAC106 | Potri.011G123500.1 | CATGGGAATTACCAGCTAAGGC | ACCCCAACTTTCTGTGTACCAC |
| NAC107 | Potri.011G149300.1 | GCCTGGGCTATCTGTGATAAAG | ATGCATGACCCAGTTTGTACGC |
| NAC108 | Potri.011G153300.1 | CTTGAGCCATGGGATATCCAAG | TGATCTTATCACGGCCAGTAGC |
| NAC109 | Potri.012G001400.1 | GAACAAGTCTGAACCCTGGGAA | TTTCTCTCCTTTAGGGGCTCTG |
| NAC110 | Potri.012G007500.1 | GTATCCAAATGGATCGAGGCAG | CAGTAGCACGATACTCATGCAC |
| NAC111 | Potri.012G023900.1 | GCATCAAGCCCTGATAAACTCC | CAGTTCCATTAGGTGAGCTTCC |
| NAC112 | Potri.012G024100.1 | CACTGGATGGTGGCTACTATGA | ACTCTGAACTCCTCTACCATCC |
| NAC113 | Potri.012G024200.1 | CTAGCGCAGGACAAGCTTTGAA | GGAGTTTATCAGGGCTTGATGC |
| NAC114 | Potri.012G038100.1 | CTACAAATGCGAGCCTTGGGAT | ATTCCGATGGCTCTATTCTGGC |
| NAC115 | Potri.012G056300.1 | GTCCACAAAGGAGGAATGGGTA | TCATGTGCGAGTCCACATTCAC |
| NAC116 | Potri.012G103500.1 | CTGCAAGTATGATCCCTGGGAA | TCAGTTCTGGATCCATGTGGAG |
| NAC117 | Potri.012G126500.1 | GCAAGACTTGATCGGAATGAGG | TCTTGAACACCCTACACACGAC |
| NAC118 | Potri.013G054000.1 | CAGTCCTAGAGATCGCAAGTAC | TAGTCTTGACACCCTTTGGAGG |
| NAC119 | Potri.013G054200.1 | CTGGTATGAGAACTAACCGTGC | CAACCCATTCATCCCTTGCTAC |
| NAC120 | Potri.013G079700.1 | CCCCAGCTTGATATCTACCAGT | CCTTTAGCTTGCCATGAACCTG |
| NAC121 | Potri.013G092400.1 | GATAAGTGCATCAGGAACACCG | TCTTGAACACTCTGCAGACCAC |
| NAC122 | Potri.013G113100.1 | CCAAGTGCAAGCTAGGGTATGA | AAGTCTGTTCTTGGTGAGCACG |
| NAC123 | Potri.014G025700.1 | CACCGATGATGTCAACGGCTAT | CCTAACCCTACCACTTATCCTC |
| NAC124 | Potri.014G041300.1 | GACATTTGGTACTTGCTTCGCG | CCAGTATTCCTGCATCAACCAG |
| NAC125 | Potri.014G064600.1 | GACTGATGTGGAGCTTGTGCTT | TCTTCCAATCAGTTCGCTCTCC |
| NAC126 | Potri.014G075900.1 | CCAAGGCTGGATACTGGAAACT | GGCTAGAATTGGTGATGAGGTC |
| NAC127 | Potri.014G076000.1 | GATGGGTGGCTATGATCACGAA | AGCCTGCTTACGATTGGAGTAC |
| NAC128 | Potri.014G076100.1 | CATGGCAGTCGTTAACCTTTGC | GCCGTTATTGACTTTTGCTGGC |
| NAC129 | Potri.014G104800.1 | CTGGACGATAGCACCAGTGATA | GTATCTGCACTGATGGGTGATG |
| NAC130 | Potri.014G107600.1 | CCTTTATCAGTGGGATCCATGG | CGCGTGATATTACGAACCTTGC |
| NAC131 | Potri.014G107700.1 | GCAGAGGCTAATGTGTGCGATT | CCAGTATCCAGCTTGAGTTGTC |
| NAC132 | Potri.014G107900.1 | CAATCGCAGAGGCTAATGTGTG | CTTGAGTTGTCCTATTAGCCCG |
| NAC133 | Potri.014G108000.1 | GCAGAGGCTAATGTGTGCGATT | GACTATACGATCCTTGCCTGTG |
| NAC134 | Potri.014G108100.1 | GAAGACTTGTCAATCATGCCGG | ACCAATCACCTTCTTGGTACCC |
| NAC135 | Potri.014G163600.1 | CTCTTCACCACCATCCATACTC | ACTCTTGATGCCATGTACCAGG |
| NAC136 | Potri.015G002900.1 | CACAAGTCAGGTGATAATGGCC | TCCAACCATCATGGTTCGGTTG |
| NAC137 | Potri.015G004100.1 | CAGGAAGTATCCAAATGGGTCG | CCTCAGTAGCACGATACTCATG |
| NAC138 | Potri.015G007000.1 | GTCAGCAACTTCCTGCTGATGT | GTCAACCGCATAGTAGCCATTC |
| NAC139 | Potri.015G020000.1 | GAACAAGTCTGAACCCTGGGAA | TTTAGGGGCTCTACCCTTGTAG |
| NAC140 | Potri.015G030200.1 | GAGTGAACTGCCAGAATAGAGC | GACTAGTGCTTGTGCATTGGCC |
| NAC141 | Potri.015G046800.1 | CCAACTGGACTTCGAACCAACA | CCTGTACTCATGCATAACCCAG |
| NAC142 | Potri.015G102100.1 | CAAGTATGATCCCTGGGAGTTG | CTTCTTCATCCCCACAACATGG |
| NAC143 | Potri.015G127400.1 | GCCATGGGATCTTCAAGAACTG | CATTCCGATCAAGTCGTGCTTC |
| NAC144 | Potri.016G027900.1 | GGTGATGGTGGCAAGGAAGTAA | CGTTCACTCTTCTTGAGCTGAG |
| NAC145 | Potri.016G055500.1 | CATGATCAAGAGCTGGTTGTGG | TCGATCAGGTCGATTCCCATTG |
| NAC146 | Potri.016G076000.1 | GTTCAATCCATGGGAGCTTCCA | GAGTATACAGAACCTGGAGAGC |
| NAC147 | Potri.016G076100.1 | GAGTGAAGACCTTAACCAGGTC | GAGCCAGTTGCTTTCCAGTATC |
| NAC148 | Potri.016G088300.1 | GGAGCTACCGAGGATAGGAAAA | GTATTCATGCATGATCCAGCCC |
| NAC149 | Potri.017G016700.1 | CCTTCATGTGGGCACCAAATCA | CTCTCCATCGATTGTAGGGATG |
| NAC150 | Potri.017G031000.1 | CTGGCCAAGAAATGCCACTTCA | TTGACATGGCTCGTAGCTTTCC |
| NAC151 | Potri.017G031300.1 | GCGTGAACCACAGGATCTTGAA | TTAAAGGCCTCTTGTTCCCCAC |
| NAC152 | Potri.017G031600.1 | CTTGTTGGGAACAAGAGGCCTT | CCCCTTGTGCTTAATCTTGCAC |
| NAC153 | Potri.017G058100.1 | CCCAACTGCAAAGTCACTGTCA | TCGCTGTAGATTGACTGTCTCG |
| NAC154 | Potri.017G063300.1 | CCTTCGAGCTTGCCAGGTAATT | CTGCTTTGTTGCTGTCAGAAGC |
| NAC155 | Potri.017G082000.1 | CAGGAACAGTGCAAGGCCAAAT | TTCATCCCTTTAGCAGCTCGAC |
| NAC156 | Potri.017G086200.1 | CAAGTGTGAGCCATGGGATTTG | CCAAGGTCTTCTTCATGCCAAC |
| NAC157 | Potri.017G104900.1 | GACTGATGTGGAGCTTGTGCTT | CCAGTGTGATATCTCCTATCCC |
| NAC158 | Potri.017G139500.1 | GAGCTACTAAAGCTGGGTACTG | CCAATCTTTGGGATGGTTGTCG |
| NAC159 | Potri.018G003800.1 | GCCATCGGTGATGTAGATCTCA | TTCCGGTAGTCTTCCAGTATCC |
| NAC160 | Potri.018G049300.1 | CAGTGCAAGGCCAAATCGAGTT | GTGATGGTTCTGCAAGAGAAGG |
| NAC161 | Potri.018G068700.1 | CAAGAGCTGATAGAGCACCTTG | GTAAGCTTTTGAAGGCCTGTGG |
| NAC162 | Potri.018G095000.1 | GTGTTACCTGCTGTGCAAGATC | CGATCCCCATTTGGATACTTCC |
| NAC163 | Potri.019G031400.1 | GTTTGATCCATGGGACTTGCCA | ATTACACCTCCAGGCGTGATTG |
| NAC164 | Potri.019G031600.1 | GTTGAGCCATGGGATTTGCCAA | GTCTTCTTCATCCCAACAAGGC |
| NAC165 | Potri.019G063000.1 | CATGAGGAGCCTTGGCAGATAT | TTTGGAGGATTTCATGCCAGGG |
| NAC166 | Potri.019G066000.1 | GGTCATTAGAGAGGTGGACTTG | GCTGTTCCTGATGCACTTATCC |
| NAC167 | Potri.019G083600.1 | CTTCAGTCACAAGGACAGGAAG | CGTCCCTTGTAGAACACTAAGG |
| NAC168 | Potri.019G099800.1 | CTGGGACAGATTCAGATGATGG | CGTGCATCACATACTTGATCCC |
| NAC169 | Potri.019G099900.1 | GATCACCTAGTCAGCAGAATCC | TTCCTGTAGCTAGCCATGAACC |
| NAC170 | Potri.T074200.1 | CACAGAGAGGCATCGATGCATA | AACAGTGTGCCCCATCTCTTTC |
