# Supplementary material for: Transcriptome analysis of salt-responsive and wood-associated NACs in Populus simonii × Populus nigra
Source: BMC Plant Biol. 2020 Jul 6;20:317. doi: 10.1186/s12870-020-02507-z (PMC7336439; doi:10.1186/s12870-020-02507-z)
Supplement: Supplementary file 2 — Additional file 2: Table S2. Homologous information of NACs from Arabidopsis thaliana and Populus simonii×P.nigra. (DOCX 27 kb) [file 12870_2020_2507_MOESM2_ESM.docx]

Supplemental Table 2 Homologous information of NACs from *Arabidopsis thaliana* and *Populus* *simonii× P.nigra*

| Common name | Gene ID | Biological function | *Populus* genes number |
| --- | --- | --- | --- |
| ANAC083, VNI2 | [AT5G13180.1](http://planttfdb.cbi.pku.edu.cn/tf.php?sp=Ath&did=AT5G13180.1) | xylem vessel formation | NAC1,9,37,81,116,142,150,151,154 |
| ANAC042,  JUB1 | [AT2G43000.1](http://planttfdb.cbi.pku.edu.cn/tf.php?sp=Ath&did=AT2G43000.1) | stress response | NAC2, 19,36,57 |
| ANAC007,  VND4 | [AT1G12260.1](http://planttfdb.cbi.pku.edu.cn/tf.php?sp=Ath&did=AT1G12260.1) | xylem formation | NAC3,34,117,143 |
| ANAC071 | [AT4G17980.1](http://planttfdb.cbi.pku.edu.cn/tf.php?sp=Ath&did=AT4G17980.1) | cell proliferation | NAC4,32,91,126,128,  129,169 |
| ANAC104, XND1 | [AT5G64530.1](http://planttfdb.cbi.pku.edu.cn/tf.php?sp=Ath&did=AT5G64530.1) | secondary wall synthesis | NAC5,30,48,79 |
| ANAC061 | [AT3G44350.2](http://planttfdb.cbi.pku.edu.cn/tf.php?sp=Ath&did=AT3G44350.2) | response to chitin | NAC6, 88 |
| ANAC032 | [AT1G77450.1](http://planttfdb.cbi.pku.edu.cn/tf.php?sp=Ath&did=AT1G77450.1) | age-dependent and stress-induced senescence | NAC7 |
| ANAC047 | [AT3G04070.2](http://planttfdb.cbi.pku.edu.cn/tf.php?sp=Ath&did=AT3G04070.2) | ethylene biosynthesis | NAC8,35,68 |
| ANAC044 | [AT3G01600.1](http://planttfdb.cbi.pku.edu.cn/tf.php?sp=Ath&did=AT3G01600.1) | response to gamma irradiation | NAC10 |
| ANAC098, ATCUC2 | [AT5G53950.1](http://planttfdb.cbi.pku.edu.cn/tf.php?sp=Ath&did=AT5G53950.1) | shoot apical meristem formation | NAC11,12,103 |
| ANAC072, RD26 | [AT4G27410.2](http://planttfdb.cbi.pku.edu.cn/tf.php?sp=Ath&did=AT4G27410.2) | response to dessication | NAC13,89,105 |
| ANAC056, ATNAC2, NARS1 | [AT3G15510.1](http://planttfdb.cbi.pku.edu.cn/tf.php?sp=Ath&did=AT3G15510.1) | regulate embryogenesis | NAC14,102,106 |
| ANAC012, NST3, SND1 | [AT1G32770.1](http://planttfdb.cbi.pku.edu.cn/tf.php?sp=Ath&did=AT1G32770.1) | secondary wall biosynthesis | NAC15 |
| ANAC014 | [AT1G33060.1](http://planttfdb.cbi.pku.edu.cn/tf.php?sp=Ath&did=AT1G33060.1) |  | NAC16, 107 |
| ANAC031, CUC3, NAC368 | [AT1G76420.1](http://planttfdb.cbi.pku.edu.cn/tf.php?sp=Ath&did=AT1G76420.1) | shoot apical meristem formation | NAC17,59 |
| ANAC074 | [AT4G28530.1](http://planttfdb.cbi.pku.edu.cn/tf.php?sp=Ath&did=AT4G28530.1) |  | NAC18, 58 |
| ANAC017, NTL7, RAO2 | [AT1G34190.1](http://planttfdb.cbi.pku.edu.cn/tf.php?sp=Ath&did=AT1G34190.1) | responses to hydrogen peroxide-mediated oxidative stress | NAC20,56,125,157 |
| ANAC002, ATAF1 | [AT1G01720.1](http://planttfdb.cbi.pku.edu.cn/tf.php?sp=Ath&did=AT1G01720.1) | response to wounding and abscisic acid | NAC21,49,55,78,90,162 |
| ANAC014, NTL2 | [AT1G33060.2](http://planttfdb.cbi.pku.edu.cn/tf.php?sp=Ath&did=AT1G33060.2) |  | NAC22,23 |
| CBNAC, NTL9 | [AT4G35580.1](http://planttfdb.cbi.pku.edu.cn/tf.php?sp=Ath&did=AT4G35580.1) | osmotic stress responses | NAC24,27,28,131,132,  133 |
| ANAC043, NST1 | [AT2G46770.1](http://planttfdb.cbi.pku.edu.cn/tf.php?sp=Ath&did=AT2G46770.1) | secondary wall thickening | NAC25,108,129 |
| ANAC069, NTL13, NTM2 | [AT4G01550.1](http://planttfdb.cbi.pku.edu.cn/tf.php?sp=Ath&did=AT4G01550.1) | response to abiotic stresses | NAC26 |
| CBNAC, NTL9 | [AT4G35580.3](http://planttfdb.cbi.pku.edu.cn/tf.php?sp=Ath&did=AT4G35580.3) | osmotic stress responses; leaf senescence | NAC29,127,130 |
| ANAC034, ANAC035, AtLOV1 | [AT2G02450.1](http://planttfdb.cbi.pku.edu.cn/tf.php?sp=Ath&did=AT2G02450.1) | control flowering time | NAC31,46 |
| ANAC029, ATNAP | [AT1G69490.1](http://planttfdb.cbi.pku.edu.cn/tf.php?sp=Ath&did=AT1G69490.1) | leaf senescence | NAC33,44,86,95,123 |
| ANAC025, TAPNAC | [AT1G61110.1](http://planttfdb.cbi.pku.edu.cn/tf.php?sp=Ath&did=AT1G61110.1) | fornormal seed development and morphology | NAC38,40,69,100,145,  148 |
| ANAC073, SND2 | [AT4G28500.1](http://planttfdb.cbi.pku.edu.cn/tf.php?sp=Ath&did=AT4G28500.1) | lignin polymerization and signaling | NAC39,82,101,149 |
| ANAC028 | [AT1G65910.1](http://planttfdb.cbi.pku.edu.cn/tf.php?sp=Ath&did=AT1G65910.1) |  | NAC41,124,158 |
| ANAC101, VND6 | [AT5G62380.1](http://planttfdb.cbi.pku.edu.cn/tf.php?sp=Ath&did=AT5G62380.1) | plant metaxylem and protoxylem vessel formation | NAC42,43,50,73,135 |
| ANAC036 | [AT2G17040.1](http://planttfdb.cbi.pku.edu.cn/tf.php?sp=Ath&did=AT2G17040.1) | drought and salt stress tolerance | NAC45,53,92 |
| ANAC103 | [AT5G64060.1](http://planttfdb.cbi.pku.edu.cn/tf.php?sp=Ath&did=AT5G64060.1) |  | NAC47 |
|  | [AT3G12910.1](http://planttfdb.cbi.pku.edu.cn/tf.php?sp=Ath&did=AT3G12910.1) | leaf senescence | NAC51,77 |
| ANAC022,NAC021, NAC1 | [AT1G56010.2](http://planttfdb.cbi.pku.edu.cn/tf.php?sp=Ath&did=AT1G56010.2) | shoot apical meristem formation and auxin-mediated lateral root formation | NAC52,76 |
| ANAC037, VND1 | [AT2G18060.1](http://planttfdb.cbi.pku.edu.cn/tf.php?sp=Ath&did=AT2G18060.1) | xylem formation | NAC54,75,153 |
| ANAC050 | [AT3G10480.2](http://planttfdb.cbi.pku.edu.cn/tf.php?sp=Ath&did=AT3G10480.2) | flowering time | NAC60,62,63,65,66,67 |
| ANAC038 | [AT2G24430.2](http://planttfdb.cbi.pku.edu.cn/tf.php?sp=Ath&did=AT2G24430.2) | shoot apical meristem formation | NAC61,74,144,159 |
| ANAC091, TIP | [AT5G24590.2](http://planttfdb.cbi.pku.edu.cn/tf.php?sp=Ath&did=AT5G24590.2) | virus basal resistance response | NAC64,110,168 |
| ANAC075 | [AT4G29230.1](http://planttfdb.cbi.pku.edu.cn/tf.php?sp=Ath&did=AT4G29230.1) | xylem formation | NAC70,161 |
| ANAC061 | [AT3G44350.1](http://planttfdb.cbi.pku.edu.cn/tf.php?sp=Ath&did=AT3G44350.1) |  | NAC71 |
| ANAC090 | [AT5G22380.1](http://planttfdb.cbi.pku.edu.cn/tf.php?sp=Ath&did=AT5G22380.1) |  | NAC72,146,147 |
| ANAC082, VNI1 | [AT5G09330.4](http://planttfdb.cbi.pku.edu.cn/tf.php?sp=Ath&did=AT5G09330.4) |  | NAC80 |
| ANAC078, NAC2, NTL11 | [AT5G04410.1](http://planttfdb.cbi.pku.edu.cn/tf.php?sp=Ath&did=AT5G04410.1) | response to high light stress | NAC83,99 |
| ANAC033, SMB, URP7 | [AT1G79580.3](http://planttfdb.cbi.pku.edu.cn/tf.php?sp=Ath&did=AT1G79580.3) | root cap development | NAC84,97,165 |
| ANAC020 | [AT1G54330.1](http://planttfdb.cbi.pku.edu.cn/tf.php?sp=Ath&did=AT1G54330.1) |  | NAC85,96 |
| ANAC008, SOG1 | [AT1G25580.1](http://planttfdb.cbi.pku.edu.cn/tf.php?sp=Ath&did=AT1G25580.1) | response to gamma irradiation | NAC87,94 |
| ANAC040, NTL8 | [AT2G27300.1](http://planttfdb.cbi.pku.edu.cn/tf.php?sp=Ath&did=AT2G27300.1) | salt-responsive flowering; gibberellic acid-mediated salt signaling | NAC93 |
| ANAC050, NAC050 | [AT3G10480.1](http://planttfdb.cbi.pku.edu.cn/tf.php?sp=Ath&did=AT3G10480.1) | flowering time | NAC98 |
| ANAC086 | [AT5G17260.1](http://planttfdb.cbi.pku.edu.cn/tf.php?sp=Ath&did=AT5G17260.1) | directing sieve element enucleation and cytosol degradation | NAC104,111,112,113,  134 |
| ANAC100, ATNAC5 | [AT5G61430.1](http://planttfdb.cbi.pku.edu.cn/tf.php?sp=Ath&did=AT5G61430.1) |  | NAC109,139,156 |
| ANAC057 | [AT3G17730.1](http://planttfdb.cbi.pku.edu.cn/tf.php?sp=Ath&did=AT3G17730.1) | directing sieve element enucleation and cytosol degradation | NAC114,120,140 |
| ANAC058 | [AT3G18400.1](http://planttfdb.cbi.pku.edu.cn/tf.php?sp=Ath&did=AT3G18400.1) |  | NAC115,141 |
| ANAC047 | [AT3G04070.1](http://planttfdb.cbi.pku.edu.cn/tf.php?sp=Ath&did=AT3G04070.1) | leaf movement and cell expansion | NAC118,163 |
| ANAC087 | [AT5G18270.1](http://planttfdb.cbi.pku.edu.cn/tf.php?sp=Ath&did=AT5G18270.1) |  | NAC119,164 |
| ANAC070,  BRN2 | [AT4G10350.1](http://planttfdb.cbi.pku.edu.cn/tf.php?sp=Ath&did=AT4G10350.1) | regulate root cap maturation | NAC121,166 |
| ANAC030, VND7 | [AT1G71930.1](http://planttfdb.cbi.pku.edu.cn/tf.php?sp=Ath&did=AT1G71930.1) | plant metaxylem and protoxylem vessel formation | NAC122,136,167 |
| ANAC062, NTL6 | [AT3G49530.1](http://planttfdb.cbi.pku.edu.cn/tf.php?sp=Ath&did=AT3G49530.1) | response to cold stress | NAC137 |
| ANAC041 | [AT2G33480.1/2](http://planttfdb.cbi.pku.edu.cn/tf.php?sp=Ath&did=AT2G33480.2) | regulate the expression of CSLA9 | NAC138,152 |
| ANAC009, FEZ | [AT1G26870.1](http://planttfdb.cbi.pku.edu.cn/tf.php?sp=Ath&did=AT1G26870.1) | control the orientation of cell division plane | NAC155,160 |
| ATWRKY19, MAPKKK11, MEKK4 | [AT4G12020.1](http://planttfdb.cbi.pku.edu.cn/tf.php?sp=Ath&did=AT4G12020.1) | disease resistance | NAC170 |
